# Institutional-Level Interventions Impacted Burnout and Compassion Satisfaction Among Medical Students During the COVID-19 Pandemic

**DOI:** 10.1007/s40670-025-02354-3

**Published:** 2025-03-08

**Authors:** Shipra Singh, Mounika Polavarapu, Camelia Arsene

**Affiliations:** 1 Department of Population Health, College of Health & Human Services, University of Toledo, Mail Stop #119, 2801 W. Bancroft Street, Toledo, OH 43606-3390 USA; 2 Department of Pediatrics, College of Health & Human Services, University of Toledo, Mail Stop #119, 2801 W. Bancroft Street, Toledo, OH 43606-3390 USA; 3 Department of Obstetrics and Gynecology, College of Health & Human Services, University of Toledo, Mail Stop #119, 2801 W. Bancroft Street, Toledo, OH 43606-3390 USA; 4 School for the Advancement of Interprofessional Education (IPE), College of Health & Human Services, University of Toledo, Mail Stop #119, 2801 W. Bancroft Street, Toledo, OH 43606-3390 USA; 5 Health Equity Research Center (HERC), College of Health & Human Services, University of Toledo, Mail Stop #119, 2801 W. Bancroft Street, Toledo, OH 43606-3390 USA; 6 Department of Medicine, College of Medicine & Life Sciences, University of Toledo and Promedica, Toledo, OH USA

**Keywords:** COVID-19 pandemic, Compassion satisfaction, Burnout, Resilience, Medical students

## Abstract

**Introduction:**

The COVID-19 pandemic presented unprecedented challenges not only to the healthcare system, but also to the medical education curricula. Institutions implemented policies regarding educational experiences for medical students in observance of the ever-changing pandemic and quarantine requirements. Additionally, medical students experienced significant stressors related to the pandemic that tested their resilience, which is the ability to bounce back when faced with adversity.

**Method:**

This cross-sectional study explored the independent relationship of pandemic-related stress and educational experience with burnout and compassion satisfaction. In addition, it also investigated the relationship of both actions-related resilience and beliefs-related resilience on burnout and compassion satisfaction. Study participants were students (*N* = 145) enrolled in a mid-western medical school during the academic year of 2020 and 2021. STATA/SE version18 was used to conduct descriptive and multivariable linear regression model statistics.

**Results:**

Results showed that among medical students, pandemic-related stress was a significant predictor of burnout (*b* = 0.31; SE = 0.05; *p* < 0.001), while a good educational experience was protective (*b* =  − 0.28; SE = 0.05; *p* < 0.001). However, resilience had no significant association with burnout. A positive educational experience was also significantly associated with higher levels of compassion satisfaction (*b* = 0.34; SE; *p* < 0.001) among the medical students. Total resilience (*b* = 0.44; *p* < 0.05) and actions-related resilience (*b* = 0.69; *p* < 0.05) independently showed a significant association with compassion satisfaction.

**Conclusion:**

Multi-dimensional interventions, including individual-level and institutional-level support, benefit individual health professionals but can also contribute to a more resilient and sustainable healthcare system capable to meeting the demands of any critical health crisis.

## Introduction

Burnout, described as a syndrome resulting from chronic exposure to workplace stress, is characterized by feelings of exhaustion, cynicism and depersonalization, and reduced compassion efficacy and accomplishment [1, 2]. Research before the COVID-19 pandemic showed that about 40–50% of healthcare providers experienced burnout across various clinical specialties [3, 4]. Compassion satisfaction is known to play a role in alleviating burnout [5]. It is described as a sense of fulfillment that comes from the joy of helping others by addressing their needs during stressful events [6].

Resilience plays an equally important role in protecting against burnout. It is a psychological phenomenon defined as one’s capacity to bounce back from adversity and is thought to protect people from stressful events and burnout [7, 8]. However, not many studies have looked at the impact of different aspects of resilience, including *beliefs* that make a person resilient and *actions* that a person takes to manage stressful situations. Numerous research studies have found that building resilience can be essential to reducing emotional distress and promoting medical training; thus, recommended adding it to the medical education curriculum [9–11].

The COVID-19 pandemic presented unprecedented challenges to healthcare systems and education. It burdened healthcare providers with increased stress in their workplace, leading to increased exhaustion and burnout [12–14]. The COVID-19 pandemic may have also affected their levels of satisfaction in their compassion capacity [15]. However, limited studies have examined factors that impact compassion satisfaction during these times of increased workplace stress [16, 17]. The medical students also faced substantial disruptions in medical education. The pandemic significantly challenged the organization, structure, and implementation of clinical education for healthcare students. Medical schools had to reassess how to incorporate clinical learning while balancing the threats of infection and its complications. Didactics that were previously in-person changed to being held virtually. However, hands-on learning, such as clinical rounding or lab-based curriculum, was often more challenging to pivot. As the structure of medical education flexed to accommodate the pandemic and quarantine, medical students reported increased challenges in their educational journey [18].

There is a significant gap in the literature regarding the long-term impact of the COVID-19 pandemic on medical education and its effects on burnout and resilience among medical students and healthcare providers. While existing studies primarily focus on immediate consequences, there is a lack of longitudinal research examining how pandemic-related disruptions influence burnout and resilience over time. Comparative analyses between pre- and post-pandemic cohorts are scarce, making it unclear whether changes in curriculum structure—such as reduced clinical exposure and increased reliance on virtual learning—have lasting implications on mental health and professional well-being. Addressing these gaps is essential for optimizing medical education and supporting the long-term well-being of future healthcare professionals.

Consequently, this research study is the first step in understanding the impact of changes in the medical education during the pandemic. Specifically, this study examined the experiences of medical students enrolled in medical school during the early pandemic years of 2020 and 2021. Specifically, it aimed to describe the relationship between stress (related to the pandemic and educational experience) and resilience on burnout and compassion satisfaction. By examining both resilience *actions* and *beliefs* in addition to total resilience, this study also aimed to better understand the distinct protective effects of each type of resilience on burnout and compassion satisfaction.

## Material and Methods

### Study Design, Participants, and Sampling

During the spring semester of 2022, a cross-sectional study was conducted among medical students who were enrolled at the University of Toledo, Toledo, OH, during the COVID-19 pandemic years of 2020 and 2021. Students who participated in the study were enrolled in the first, second, third, and fourth academic years (M1, M2, M3, M4) of their medical school training. The academic curriculum in the first 2 years of medical school mostly includes predominantly didactic training, with increasing clinical electives in their third and fourth years. The research protocol was approved by the Institutional Review Board (IRB #301,688). The institution’s Department of Medical Education facilitated the dissemination of the recruitment email among medical students willing to participate in the study.

### Survey Instrument

The survey instrument was built using the Qualtrics online survey software. The face validity of the instrument was established via a comprehensive review of the published literature, and content validity was established by having the survey reviewed by four external experts. The Cronbach alpha values ranged from 0.72 to 0.91 for the scales on the survey.

### Measures

The survey included items regarding demographic details, physical health, mental health, educational experience, pandemic stress, resilience (total, action, and belief), burnout, and compassion satisfaction.

#### Burnout

Burnout was adapted from the Compassion Quality of Life Scale (ProQOL) version V scale with one minor modification [19]. The original word ‘helper’ was replaced with ‘provider’ to better match the medical students’ scenarios. Burnout was measured using 10 questions inquiring about the frequency of various emotions related to burnout, with five possible responses: 1 (never), 2 (seldom), 3 (sometimes), 4 (often), and 5 (always). Examples of items included were “I feel worn out because of my work as a provider,” “I am not productive at work because I am losing sleep over traumatic experiences of a person for whom I was a provider,” and I feel bogged down by the system.” The Cronbach’s alpha for the scale in our study was 0.86. The potential range for burnout was 10–50, and higher scores indicated greater levels of burnout.

#### Compassion Satisfaction

Compassion satisfaction was adapted from the Compassion Quality of Life Scale (ProQOL) version V scale with one minor modification [19]. The original word ‘helper’ was replaced with ‘provider’ to better match the medical students’ scenarios. Compassion was evaluated using 10 questions inquiring how frequently the medical students experienced various emotions related to compassion satisfaction, with five possible responses: 1 (never), 2 (seldom), 3 (sometimes), 4 (often), and 5 (always). Examples of items included were “I believe I can make a difference through my work” and “I get satisfaction from being able to be a provider to people”. The Cronbach’s alpha for this scale was 0.95. The potential range for compassion satisfaction was 10–50, and higher scores reflected greater levels of compassion satisfaction.

#### Resilience

The Brief Resilient Coping Scale (BRCS) was used to assess the total resilience in the study [20]. This 4-item scale assesses the resourcefulness, control, growth, and involvement domains of resilience. Responses are scored on a 5-point Likert scale ranging from 1 = does not describe you very well to 5 = describes you at all. The Cronbach’s alpha for the scale in our study was 0.72. The overall range for total resilience was 4–10, with higher scores indicating better resilience. Next, the BRCS items were further grouped into *resilience action* that summed the following two items—“I look for creative ways to alter difficult situations” and “I actively look for ways to replace the losses I encounter in life”—and *resilience belief* that summed the following two items—“Regardless of what happens to me, I believe I can control my reaction to it” and “I believe I can grow in positive ways by dealing with difficult situations.” The range for each action resilience and belief resilience subscales were 2–10, with higher scores indicating better action and belief resilience.

#### Educational Experience

The Higher Education Stress Inventory (HESI) scale was used to assess the perception of education experiences of medical students during the pandemic. This scale has been extensively used to describe several stressful aspects of higher education among medical and nursing students [21]. This 33-item instrument consists of seven domains related to educational stress: worries about future endurance/competence, faculty shortcomings, nonsuppurative climate, workload, insufficient feedback, lack of commitment, and financial concerns. Responses are scored on a 4-point Likert scale ranging from 1 = totally disagree to 4 = totally agree. The Cronbach’s alpha for the scale in our study was 0.88. The range for educational experience was 33–132, and higher scores represented a more positive educational experience.

#### Pandemic Stress

The modified version of the Perceived Stress Scale-10 (PSS-10) related to COVID-19 (PSS-10-C) was used to measure pandemic stress [22]. The PSS-10-C is made up of 10 items that include items such as “I have felt that I am unable to control the important things in my life because of the epidemic”; “I have felt that the difficulties are increasing in these days of the epidemic, and I feel unable to overcome them”; and “I have felt optimistic that things are going well with the epidemic.” Responses are scored on a 5-point Likert scale with five response options: never, almost never, occasionally, almost always, and always. The Cronbach’s alpha for the scale in our study was 0.91. The range for pandemic stress was 10–50, with higher scores representing greater stress due to the pandemic.

#### Other Predictor Variables

This included assessment of age, sex, race, ethnicity, year of starting medical school (2017–2019 and 2020–2021), overall physical health, and overall mental health.

### Data Collection

Following Institutional Review Board approval, data collection was done during the 2022 spring semester using an anonymous survey link sent to the medical student emails. Students were given access to an electronic informed consent prior to accessing the survey. The consent form clearly explained the study’s purpose, procedures, potential risks, and benefits, ensuring participants fully understood what participation entailed. Participants were informed that their participation was voluntary and that they could withdraw at any time without any repercussions. All data were securely stored in encrypted digital files on password-protected servers, accessible only to the research team. After completion of the survey, students were offered an opportunity to enter a random drawing for one $50 e-gift card.

### Data Analysis

Data analyses were performed using STATA-SE version 18 [23]. Descriptive statistics were used to characterize the survey respondents using frequencies and percentages for categorical variables and means and standard error for continuous variables. Next, multivariable linear regression was conducted to analyze the independent associations of total resilience, resilience action, and resilience belief on burnout among medical students after controlling for sociodemographic variables. A similar multivariable linear regression was conducted to analyze the independent associations of total resilience, resilience action, and resilience belief on compassion among medical students after controlling for sociodemographic variables.

## Results

Table 1 summarizes key information in regard to the sociodemographic characteristics of the total sample of 145 medical students (57.24% of participants were females and 66.9% identified as White race). The table also presents the means and standard errors for the sample in regard to physical health, mental health, educational experience, pandemic stress, resilience, burnout, and compassion satisfaction. Mean scores of burnout showed moderately high-level burnout and mean scores of pandemic stress showed high level of stress experience; however, mean scores for compassion satisfaction showed high levels of compassion reported by students. The mean scores of educational experience conveyed positive perception of educational experience. Overall resilience mean scores showed high total resilience, with higher belief resilience than action reported. The response rate for our study was 20.7%.

**Table 1:** Description of key variables in the study

Variable	*N* (%)	Mean (SE)	Range (min–max)
Sex			
Male	62 (42.76%)		
Female	83 (57.24%)		
Race			
White	97 (66.9%)		
Others	48 (33.10%)		
Medical school start year			
During 2017–2019	42 (28.97)		
During 2020–2021	103 (71.03)		
Physical health		2.02 (0.06)	
Mental health		2.26 (0.07)	
Educational experience		83.8 (0.83)	33–132
Pandemic stress		27.86 (0.61)	10–50
Resilience total		14.94 (0.23)	4–20
Resilience action		6.99 (0.13)	2–10
Resilience belief		7.96 (0.14)	2–10
Burnout		25.43 (0.56)	10–50
Compassion satisfaction		38.63 (0.60)	10–50

Table 2 presents the three independent multivariable regression models that were assessed to understand burnout among medical students during the COVID-19 pandemic. Along with the sociodemographic characteristics of sex, race, and the start year of medical school, each model included physical health, mental health, educational experience, and pandemic stress as predictors. Resilience was measured as total resilience (action + belief) in model 1, only as action in model 2, and only as belief in model 3. The results indicated that educational experience was consistently and negatively associated with burnout across all models (*b* =  − 0.28; SE = 0.05; *p* < 0.001). Pandemic stress was significantly and positively associated with increased burnout among medical students after adjusting for the remaining covariates. Resilience, measured as total resilience in model 1, action in model 2, and belief in model 3, did not show significant associations with burnout in this sample. The adjusted *R*^2^ values indicated a strong and statistically significant model fit, with model 1 explaining 55.55% of the variance in burnout, model 2 at 55.61%, and model 3 at 55.28%.

**Table 2:** Independent multivariable regression of total resilience, resilience action, and resilience belief on burnout among medical students (*N* = 145)

Variable	Model 1 *B* coeff (SE)	Model 2 *B* coeff (SE)	Model 3 *B* coeff (SE)
Sex			
Male	Ref	Ref	Ref
Female	− 1.26 (0.79)	− 1.25 (0.79)	− 1.21 (0.79)
Race			
White	Ref	Ref	Ref
Others	0.53 (0.81)	0.58 (0.81)	0.45 (0.81)
Medical school start year			
During 2017–2019	Ref	Ref	Ref
During 2020–2021	− 0.36 (0.85)	− 0.36 (0.85)	− 0.33 (0.85)
Physical health	1.17 (0.59)	1.13 (0.59)	1.25* (0.59)
Mental health	0.22 (0.58)	0.26 (0.58)	0.23 (0.58)
Educational experience	− 0.28** (0.05)	− 0.28** (0.05)	− 0.28** (0.05)
Pandemic stress	0.31** (0.06)	0.32** (0.06)	0.31** (0.07)
Resilience total	− 0.18 (0.17)		
Resilience action		− 0.31 (0.26)	
Resilience belief			− 0.19 (0.28)

*p < 0.05; **p < 0.001; *Ref*, reference

Table 3 presents the impact of various factors on compassion satisfaction among medical students using three independent multivariable regression models. Model 1 incorporated total resilience (action + belief) as a predictor, model 2 included resilience action, and model 3 factored in resilience belief while adjusting for sex, race, start year of medical school, physical health, mental health, educational experience, and pandemic stress. Educational experience displayed a consistently positive and statistically significant association across all three models (*b* = 0.34 to 0.36; *p* < 0.001) with compassion satisfaction among medical students in the study. Pandemic stress showed a positive but not statistically significant association with compassion satisfaction (*b* = 0.06 to 0.08). Regarding resilience, the total resilience score in model 1 (*b* = 0.44; *p* < 0.05) and resilience action in model 2 (*b* = 0.69; *p* < 0.05) both showed positive and statistically significant associations with compassion satisfaction after controlling for the remaining covariates. The adjusted *R*^2^ values indicated a moderate explanation of variance in compassion satisfaction by the models, with the value being 32.47% for model 1, 32.32% for model 2, and 31.42% for model 3.

**Table 3:** Independent multivariable regression of total resilience, resilience action, resilience belief on compassion satisfaction among medical students (*N* = 145)

Variable	Model 1 *B* coeff (SE)	Model 2 *B* coeff (SE)	Model 3 *B* coeff (SE)
Sex			
Male	Ref	Ref	Ref
Female	1.56 (1.05)	1.51 (1.05)	1.45 (1.05)
Race			
White	Ref	Ref	Ref
Others	0.07 (1.07)	− 0.02 (1.01)	0.28 (1.07)
Medical school start year			
During 2017–2019	Ref	Ref	Ref
During 2020–2021	1.16 (1.12)	1.15 (1.12)	1.11 (1.13)
Physical health	− 0.34 (0.78)	− 0.28 (0.79)	− 0.53 (0.78)
Mental health	− 0.83 (0.77)	− 0.98 (0.76)	− 0.82 (0.78)
Educational experience	0.34** (0.07)	0.36** (0.06)	0.35** (0.07)
Pandemic stress	0.07 (0.09)	0.06 (0.09)	0.08 (0.09)
Resilience total	0.44* (0.21)		
Resilience action		0.69* (0.34)	
Resilience belief			0.55 (0.37)

*p < 0.05; **p < 0.001; *Ref*, reference

## Discussion

This study showed that COVID-19 pandemic–related stress was a significant predictor of burnout among medical students, while a good educational experience was protective. Higher resilience did not protect medical students against burnout. A positive educational experience was also significantly associated with higher levels of compassion satisfaction. Finally, better overall resilience, specifically resilience characterized by action-oriented behaviors, was significantly associated with greater compassion satisfaction among medical students.

### Burnout Among Medical Students

Pandemic stress emerged as a significant predictor of burnout among medical students. The coefficient for pandemic stress was positive in all three models, suggesting that higher levels of stress related to the pandemic were significantly correlated with increased burnout among medical students after adjusting for the remaining covariates. Studies have shown that the prevalence of burnout among physicians increased during the pandemic years [14, 24, 25]. Successful pre-pandemic workplace interventions were insufficient to address the magnitude of challenges [5]. Stressors such as disruption of in-person learning, limited clinical exposure, and the abrupt transition to online education led to uncertainty and frustration among medical students [6]. While institutional attention during the pandemic focused primarily on meeting the escalated needs for physicians’ social and emotional support, burnout among medical students received comparatively less attention. Our study results reported that pandemic-related stress significantly impacted burnout among medical students, even when accounting for individual coping mechanisms such as resilience.

While resilience is often highlighted as a buffering mechanism against burnout [7], our findings challenge this perspective. In our study, resilience did not demonstrate a statistically significant relationship with burnout after controlling for other variables, including sex, race, start year of medical school, educational experience, pandemic-related stress, and health status. Therefore, in the context of a crisis such as a pandemic, identifying specific protective factors to mitigate the risk of burnout for medical students is essential. Furthermore, our study showed that educational experience during the pandemic was critical in predicting burnout, suggesting that a positive perception of the educational experience by medical students was protective against burnout. These perceptions of the educational experiences of the medical students were significantly determined by the institutional policies and interventions that were implemented to address the challenges during the pandemic [26]. However, prior research has mostly investigated the role of sociodemographic characteristics of medical students on burnout. For example, during the pandemic, a study found that changes in modality of instruction to distance learning and its related stress varied across different demographic groups [27], while a meta-analysis reported that female students were more likely to experience burnout [28].

Our study’s results are very similar with those from other studies where health system–level institutional strategies that impact educational experiences and decrease burnout due to the increased quality of learning, such as providing flexible scheduling, engaging virtual learning, multiple learning accessible resources, and opportunities for peer support, have been shown to reduce educational stress more effectively among medical students during the COVID-19 pandemic [29]. Other specific educational interventions that worked well for our students were based on using innovative technology methods in medicine such as involving students under the supervision of mentors like residents and faculty in telemedicine visits; project-specific learning with projects based on collecting data remotely through chart review or virtual surveys for public-health COVID-10 initiatives or other specialty-specific projects; and creating health resources for patients and the community. Our results suggest that institutional-level support promoting flexible and engaging educational environments could provide greater protection against burnout than individual-level sociodemographic factors.

### Compassion Satisfaction Among Medical Students

Our study finding suggested that an increase in the educational experience score (implicating positive educational experience) correlated with higher levels of compassion satisfaction. These findings share similarities with existing literature on compassion fatigue among healthcare providers, with distinctions based on their professional context. For medical students, institutional policies that created a positive educational experience had a significant impact on improving compassion satisfaction. Health systems that engaged providers in leadership positions and departments that implemented strategies to address stress and enhance a positive work environment were found to have providers with higher compassion satisfaction [30]. Furthermore, moral injury from difficult decision-making during the COVID-19 pandemic significantly impacted compassion satisfaction among healthcare providers, which impacted their overall well-being and compassion fatigue [31, 32]. Thus, implementing flexible sessions for providers, supporting nontraditional hours for work, and short-term interventions to promote mental health are critical strategies to address compassion fatigue [31].

For medical students, academic pressures, limited clinical experience, and the stress of transitioning into clinical roles are primary contributors to educational stress [8]. Our findings build on this understanding, showing that institutional policies aimed at creating a good educational experience can help promote compassion satisfaction. Additionally, our study found that overall resilience and action-oriented resilience were significantly related to greater compassion satisfaction among medical students. These results suggest that higher levels of resilience, whether in total or specifically through action-oriented behaviors, are related to greater compassion satisfaction among participating medical students. Resilience belief in model 3, although not statistically significant, also showed a positive association, which may indicate a trend that warrants further investigation. Individuals with greater resilience usually utilize problem-solving strategies and seek social support to manage workplace stress which may lead to a decrease in compassion fatigue [33, 34]. Based on the Brief Resilience Coping Scale, belief-based resilience represents a cognitive approach to stress, while action-oriented resilience shows a behavioral and problem-solving approach. To further explain these concepts: belief-based resilience refers to having confidence in one’s ability to overcome difficulties and viewing setbacks as opportunities for personal growth; action-oriented resilience refers to taking action, asking for help, being positive, and having a sense of purpose. Our study is the first to examine their impact separately on compassion fatigue among medical students. Our findings suggest that identifying effective training and strategies to facilitate action-oriented resilience among medical students is more beneficial.

### Strengths and Limitations of the Study

The primary limitation of our study includes the cross-sectional research design that restricts the ability to establish any causation between the key variables assessed. Second, the reliance on self-reported data may introduce response biases, as participants may have answered questions in a socially desirable manner rather than reflecting their true beliefs and behaviors. Another limitation is the fact that the ‘educational experience’ measurement is ‘the perception of educational experience,’ as this is a single-institution study where all participants likely received the same standard of education, and so the metric is evaluating the participants opinions/perceptions of their educational quality as opposed to differences between participants in what, how, and when they were taught. The results from our regional study may also not be generalizable to the experiences of medical students in other demographic or educational settings. Additionally, the low response rate may also limit the generalization of our findings to the broader population. Finally, the survey was conducted in spring semester of 2022 and not during the initial years of the pandemic when the most significant stressors had an impact on education. Even though data collection in this time period facilitated us to survey students enrolled in all 4 years of medical school who had experienced the pandemic during their academic years, this recruitment time could potentially influence the results of the study in regard to resilience and burnout. However, a significant strength was the utilization of the measures used in the survey that reported high Cronbach alpha values ranging from 0.72 to 0.91 for all the scales, indicating a strong internal consistency and reliability.

## Conclusion

In conclusion, examining core concepts of resilience, stress, burnout, and compassion satisfaction among healthcare professionals is essential, particularly considering the challenges posed by the recent global pandemic. These concepts are fundamental in understanding the overall physical, psychological, and emotional well-being of professionals working on the frontline during such a crisis that may have exacerbated existing stressors and presented unprecedented challenges. They may have far-reaching consequences for medical students, who are still in the formative stage of their careers.

This study answered some of these questions and provided insight into areas for future interventions. It showed that stressors related to the COVID-19 pandemic were likely to predict burnout among medical students. However, students were more likely to display compassion satisfaction if they had a positive educational experience and better overall resilience, specifically through action-oriented behaviors. Furthermore, cultivating compassion satisfaction can enable medical students to find meaning and fulfillment in their work despite its challenges.

Thus, this study sheds light on how supportive institutional policies, such as frequent changes to instructional methodologies and adaptation to clinical education, impact not just the quality of education but also the mental health and professional development of medical students. Multi-dimensional intervention strategies, including individual- and institutional-level support, may not only benefit individual health professionals but can also contribute to a more resilient and sustainable healthcare system capable of meeting the demands of any critical health crisis.

Beyond the pandemic, medical schools need to develop alternative and flexible strategies that can include changes in early years curriculum by including social and population sciences, ensuring students are better prepared when they transition from undergraduate medical education to graduate medical education, developing new competencies such as those in relation to telemedicine or changing their grading and ranking approach that increase burnout and decrease teamwork to support programmatic assessments where continuous mentorship and feedback is provided. Another important issue is to make sure that they offer the best well-being and mental health support for all the students at all stages of their training. Also, these institutions need to be prepared when needed to relocate funding and have the human capital and resources to do so.
